# Reply to “Letter to the Editor”: Prognostic analysis of *Pneumocystis jirovecii* pneumonia in patients with systemic vasculitides: a retrospective cohort study

**DOI:** 10.1007/s10067-024-07174-1

**Published:** 2024-10-05

**Authors:** Ruxuan Chen, Hui Huang

**Affiliations:** Department of Pulmonary and Critical Care Medicine, Dongcheng District, Peking Union Medical College Hospital, Chinese Academy of Medical Sciences & Peking Union Medical College, #1 Shuaifuyuan Street, Beijing, 100730 China

To the Editor,

We appreciate the opportunity to respond to Han et al.'s comments on our recent article [1], **“Prognostic analysis of Pneumocystis jirovecii pneumonia in patients with systemic vasculitides: a retrospective cohort study”**, in their “Letter to the Editor”.

We agree with Han et al. [2] that when the events per variable (EPV) is smaller than 10, the effect of overfitting is pronounced. However, due to the retrospective nature and the relatively small sample size of our single-center study, we cannot increase the sample size in our study to fit the number of predictors. We mentioned this limitation in the discussion section.

Common strategies to reduce the number of predictors before developing a risk prediction model include univariable screening and stepwise model selection [3]. As we mentioned in our manuscript, the statistically significant variables selected via univariate analysis were subsequently assessed by multivariate analysis. We used stepwise backwards elimination in multivariate Cox regression analysis. In our original manuscript, we listed 11 variables with a *p* value < 0.05 in Table 2. The *p* values of CD4/CD8 ratio, urea, lactate dehydrogenase, and ferritin were > 0.05 in univariate Cox regression analysis, and these variables were not included in Table 2. During the peer review process, the reviewers suggested to assess the impact of mechanical ventilation and activity of systemic vasculitis on the survival of these patients. We agree that these are factors with important clinical significance. Therefore, we added these two variables in Table 2. The *p* value of “stable vasculitis” is closer to 0.05, so we included stable vasculitis in multivariable analysis. The *p* value of “use of invasive mechanical ventilation” is 0.319, and it was not included in multivariable analysis. We should have elaborated these details in our manuscript. Thank you for bringing up this question.

The results of multivariable Cox regression analysis suggested that microscopic polyangiitis (MPA), concomitant aspergillosis, and higher D-dimer at the diagnosis of *Pneumocystis jirovecii* pneumonia (PJP) were independent adverse prognostic factors for overall survival, while stable disease activity of vasculitis was an independent favorable prognostic factor. These results were accordant with clinical perception, except for MPA, which had not been paid enough attention in previous reports. We presented our results to call physicians’ attention to these potential prognostic factors. We agree that the results should be interpreted judiciously. We hope to verify our findings in future larger-scale prospective studies.                                                                                                                                             

## Data Availability

The datasets generated during and/or analyzed during the current study are available from the corresponding author on reasonable request.
